# Effects of high light intensity and spectral variability on maize photosynthesis and growth

**DOI:** 10.3389/fpls.2025.1511768

**Published:** 2025-03-26

**Authors:** Isabell Pappert, Celine Ühlein, Luca Jokic, Ralf Kaldenhoff

**Affiliations:** Department of Applied Plant Sciences, Faculty of Biology, Technical University of Darmstadt, Darmstadt, Germany

**Keywords:** *Zea mays*, gas exchange, chlorophyll fluorescence, photosynthesis - light conditions, photosynthesis and growth

## Abstract

This study investigates the effects of ultra-high light intensities and varying light spectra on the photosynthetic efficiency and growth of maize (*Zea mays saccharata*). Photosynthetic rates, transpiration, stomatal conductance, and leaf temperature were measured under white light, monochromatic light, and their combinations. Assimilation rates increased with light intensities up to 5000 PAR, plateaued around 5500 PAR, and declined beyond 8000 PAR. Red light at 300 PAR yielded the highest assimilation rate under monochromatic conditions, while green light significantly boosted assimilation at higher intensities, peaking at 33.5 µmol m^–2^s^–1^ under 4000 PAR. A 50% mix of white and green light at 2000 PAR enhanced assimilation by 14% compared to white light alone. Red light (630 nm) notably promoted photosynthesis in high PAR combinations. However, increasing green light reduced quantum yield, and higher blue light enhanced non-photochemical quenching. These findings suggest that ultra-high light intensities with specific spectral combinations can optimize photosynthesis in maize, though this does not necessarily translate to enhanced overall plant growth.

## Introduction

### Background on photosynthesis and light intensity

Light intensity or photosynthetically active radiation is connected to the rate of photosynthesis directly: the more light present, the higher the rate until saturation. At even higher intensity photoinhibition may occur (McCree, 1971). The quality of light, specifically its wavelength, is a critical factor in photosynthesis, as chlorophyll pigments absorb most efficiently in the blue (450–495 nm) and red (620–700 nm) light ranges (Lichtenthaler and Buschmann, 2001). Further studies examined the optimization of light spectra and intensity as means of maximizing photosynthesis rates. Studying these relationships is required to optimize photosynthetic performance under the different light conditions and comprehend the processes of photosynthetic mechanisms (Taiz et al., 2015).

### Current challenges and knowledge gaps

Understanding the response and effects of ultra-high light intensities on photosynthesis remains elusive to researchers. Destructive effect of excessive light on photochemical efficiency by photoinhibition damages the photosynthetic apparatus, hence limiting the general photosynthetic capacity (Demmig-Adams and Adams, 1992). Yet, it is not fully understood how variation of light spectra affect photosynthesis. In particular, how the different wavelengths interrelate to influence photosynthetic efficiency in maize plants. While high photosynthetic efficiency from LED light sources has been shown with red and blue light, the effect of other wavelengths is quite undetermined (Genty et al., 1989). Similarly, ultra-high light intensities in combination with different light spectra and their interaction on photosynthetic processes were not investigated in detail. Hence, leaving a knowledge gap regarding optimization of light conditions toward maximum photosynthetic performance (Hikosaka and Terashima, 1995).

### Importance of the study

Maize (*Zea mays*) is one of the most important crops, serving as a staple food, livestock feed, and raw material for biofuels and industrial products (Ranum et al., 2014). Its C4 photosynthetic pathway makes maize highly efficient in utilizing light and carbon dioxide under optimal conditions, giving it a key role in addressing global food security (Sage and Zhu, 2011). Thus, it is important to understand how light and high light intensity as well as the change in light spectra act on maize. Already today and in the nearest future controlled-environment agriculture featuring artificial lighting as a key input for maximum yield becomes more important (Malabadi Rb et al., 2024). Maize was used in this study as a model to study the answer of plants with C4 photosynthesis. The results presented here support strategies towards optimization of photosynthetic efficiency not only in maize but also in other important crops that share similar photosynthetic pathways.

### Objective of the study

With this study, we try to improve our understanding to this regard and also concerning photosynthetic efficiency under varying light intensities. We observed photosynthetic responses in maize (*Zea mays saccharata*) exposed to ultra-high light intensity conditions and varying light spectra. It provides conditions of optimal light utilization and photosynthesis efficiency.

## Material & methods

### Plant growth conditions

Maize seeds were immersed in water for 3 hours and twenty seeds were placed in a 1 L pot for germination. After 5 days seedlings were transferred to 1 L pots with a diameter of 14 cm and cultivated for 20 days until they reached the desired age for gas exchange or chlorophyll fluorescence measurements. The plants were grown in a greenhouse with a 12-hour day and night cycle at light intensity of 300 µmol m^–2^s^–1^, room temperature of 21°C, and humidity of 68%. Starting from day 10 after sowing, specific plants were exposed to green light with 2000 PAR.

### Experimental conditions and illumination

Gas exchange and chlorophyll fluorescence was determined in the youngest fully expanded leaf of 20-day old plants.

**Figure 1:**
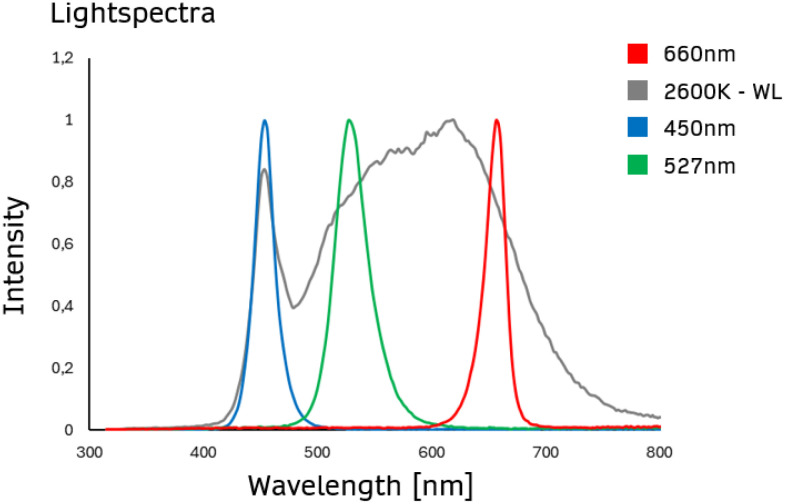
Light spectra employed to examine the effect of ultra-high light intensity on maize photosynthesis and growth. The spectra of white light (2600 K - WL), blue light (450 nm), green light (527 nm), or red light (660 nm) are presented, recorded using the SpectraPen mini (Photon Systems Instruments, Czech Republic).

In our study, plants were illuminated using Sevengines chips from Chips4Light GmbH (Regensburg, Germany) (**Figure 1**), a highly efficient LED source with a narrow viewing angle of +/- 10°. These innovative modules use total-internal-reflection (TIR) technology, which minimizes light loss and achieves a highly focused light output, making them particularly effective for precision lighting in photosynthesis research. Additionally, their customizable wavelength range (367 nm-940 nm) and optimized thermal management allow for fine-tuned light conditions, essential for exploring the effects of specific light spectra on photosynthesis and plant growth.

### Spectrum information

### Measurements and calculations

#### Steady state gas exchange measurements

We measured gas exchange in maize plants using a portable gas-exchange system (GFS-3000, Heinz Walz GmbH, Germany). The GFS-3000 gas analyzer system was used for measurement of gas exchange parameters such as the assimilation rate, transpiration rate, and stomatal conductance.

The conditions were set to 400 ppm CO_2_ concentration, 18000 ppm humidity, 25°C cuvette temperature, and 0 to 8500 µmol m^–2^s^–1^. The plants underwent a dark adaptation for 16 hours (overnight). For light response curve measurements, they were subjected to 75 minutes of 100 PAR white light to initiate photosynthesis. Subsequently, the light intensity was increased, and plants were left for 12 minutes to acclimate to each higher level of irradiation. Data were evaluated by calculating the average value of the last 2 minutes for each level of irradiance and plotting it as a function of PAR level. To compare the gas exchange parameters at 2000 PAR with different light compositions, a single 75 min measurement was carried out on a dark-adapted plant.

#### Steady state chlorophyll fluorescence measurements

The plants were dark-adapted for 60 minutes prior to treatment with the lowest light intensity of 100 PAR for 60 minutes. Each higher level was applied for 15 minutes to allow the plant to adapt to the increased light intensity and reach a steady state. **Supplementary Figure 12** presents measurement data illustrating the reaching of steady state during the measurements.

The light-dose response curves we used for our evaluation were always recorded with increasing intensity on one plant (‘LRC’ = ‘Light response curve’). Additionally, we ran trials where a new dark-adapted plant was measured for every increase in light intensity (‘SPM’ = ‘single plant measurement’). **Supplementary Figure 11** illustrates how these two methods of data collecting differ from one another.

We calculated the chlorophyll fluorescence parameters: maximal quantum efficiency of PSII [Fv/Fm], quantum yield of photosynthetic electron transport [ФPSII = (Fm’ – F )/Fm’], electron transport rate [ETR = ФPSII * PAR * 0.84 * 0.5] and quantum yield of NPQ-related energy loss [YNPQ = (F/Fm’) – (F/Fm)] from the Chl fluorescence measurement.

#### Plant morphology and health

Plants that grew under different light conditions were divided into groups with different irradiances from day 10 after sowing. From then on, all plants were scanned daily with the Multispectral 3D scanner for plant phenotyping PlantEye F600 from Phenospex (Heerlen, Netherlands).

#### Measurement repetitions

For all chlorophyll and gas exchange experiments, 10 plants per condition were examined. When examining multiple conditions consecutively on a single plant, the order of the examined conditions was always randomized.

For the design of the experiment, 20 plants per light condition were examined. For the growth experiments, 3 plants per growing condition were cultivated, i.e. a total of 9 plants. Together, a total of 420 plants were analyzed for this study.

## Results

### Photosynthetic response to varying white light intensities

**Figure 2:**
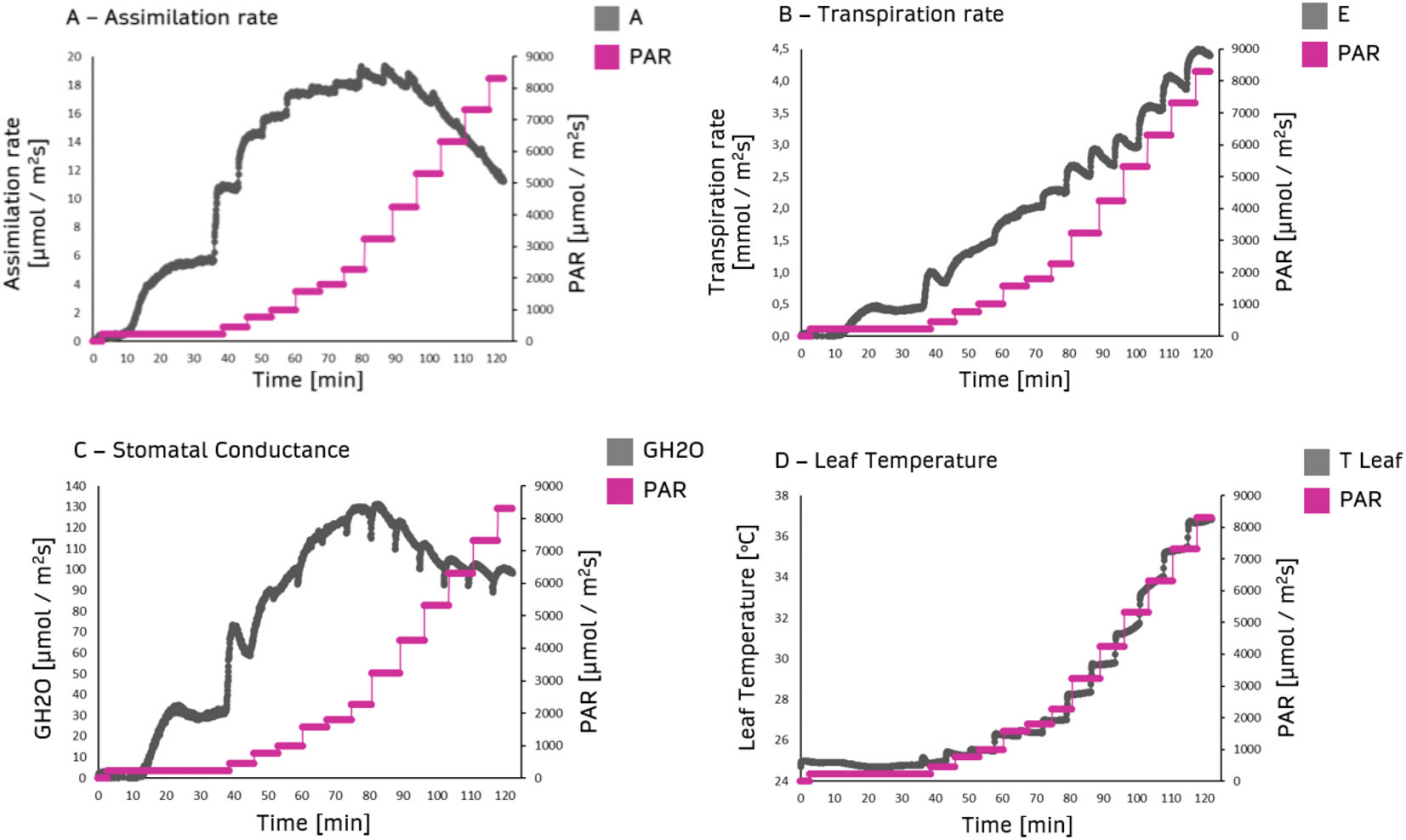
Primary ordinate: gas exchange parameters from dark-adapted plants as a function of time (abscissa). The data were obtained by measuring steady-state gas exchange using the GFS-3000 (Heinz Walz GmbH, Germany) on 20-day-old maize plants (Zea mays saccharata). First stage of the Light Response Curve was set to 75 min. All further stages 12 min. Secondary ordinate: Gradual PAR level from 0 to 8500 PAR (black curves). **(A)** Assimilation rate, **(B)** Transpiration rate, **(C)** Stomatal conductance, **(D)** Leaf temperature.

Assimilation rate, transpiration rate, stomatal conductance, and leaf temperature were assessed under different white light intensities. The assimilation rate increased to 2000 PAR irradiation intensity reaching a plateau at about 3000-4000 PAR, which was the onset of photosynthesis light-saturated phase. Beyond 4000 PAR, a decrease in assimilation rate was observed (**Figure 2A**). Transpiration rate increased with light intensity due to stomatal opening (**Figure 2B**). As expected, stomatal conductance was correlated to light intensity (**Figure 2C**). Leaf temperature was initially constant and significantly increased above 1000 PAR up to more than 36°C under 8000 PAR (**Figure 2D**).

### Gas exchange in maize under high white, red, blue, or green light

**Figure 3:**
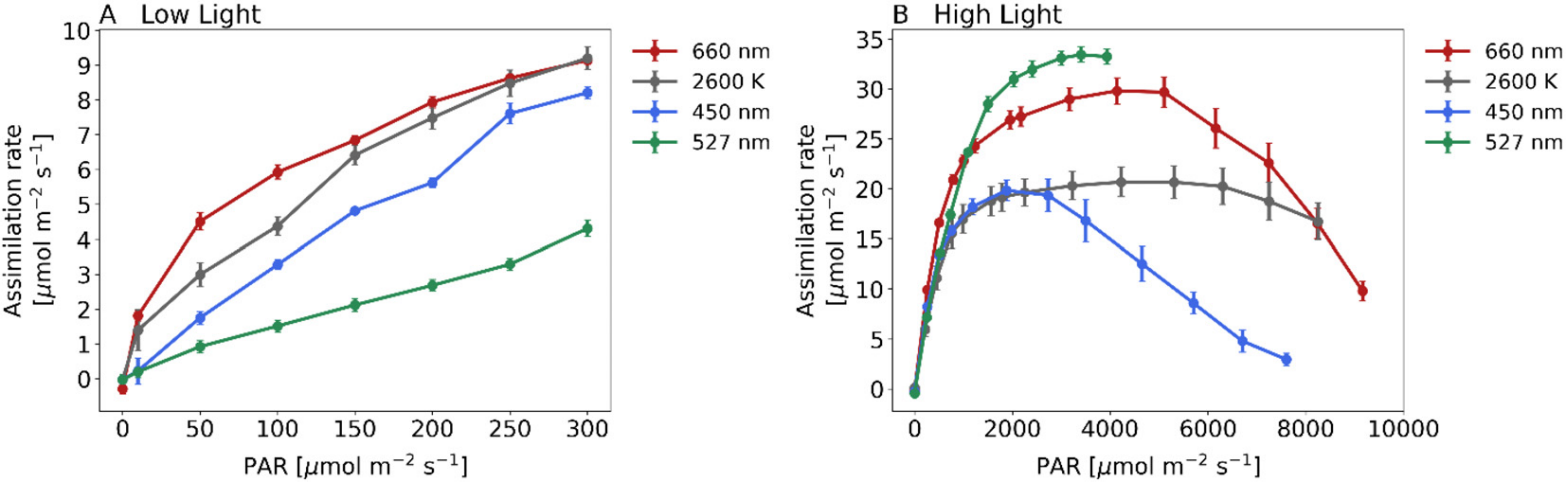
Assimilation rate for 4 different light treatments when recording steady state light response curves with the GFS-3000 (Heinz Walz GmbH, Germany) and 20-day-old dark-adapted maize plants (Zea mays saccharata). Abscissa: PAR-Level [µmol/m^2^s]. First stage of the Light Response Curve was set to 75 min. All further stages were 12 min respectively. Red curve: 660 nm, gray curve: 2600 K, blue curve: 450 nm and green curve: 527 nm. For light spectra, see **Figure 1**. Number of samples: n=10 for each light treatment.

Initially we examined the assimilation rate of plants under different light intensities, ranging from 0 to 300 PAR (**Figure 3A**). Maize plants exposed to white or red light at 300 PAR had an assimilation rate of approximately 9.2 µmol m^–2^s^–1^. Under blue light, it was 8.2 µmol m^–2^s^–1^, and under green light, it was 4.3 µmol m^–2^s^–1^. The curve remained constant within this range and without photoinhibition. We computed the slope of the curves at low PAR values to assess photosynthesis effectiveness under light-limited conditions. The mean slope of the curves was 0.053 µmol m^–2^s^–1^ for red light, 0.044 µmol m^–2^s^-1^ for white light, 0.028 µmol m^–2^s^–1^ for blue light, and 0.015 µmol m^–2^s^–1^ for green light.

Assimilation rates at higher irradiation levels are shown in **Figure 3B**. Under white light, maize plants reached the maximum light absorption capacity, the light saturation point, at about 4700 PAR. The curve remains steady at a value of approximately 16.0 µmol m^–2^s^–1^ even if the plant was exposed to 8000 PAR. Maize under red light reached the light saturation point at approximately 4500 PAR, and at around 2300 PAR under blue light. This curve has a substantial decline from approximately 3000 PAR and reaches a minimum level at 8000 PAR (2.9 µmol m^–2^s^–1^). Lastly, we examined the assimilation rate under green light, which we successfully quantified up to 4000 PAR in this study. The curve exhibits the steepest incline within the range of PAR values higher than 1000 and attains the highest rate of assimilation among the compared light conditions, with values reaching up to 33.5 µmol m^–2^s^–1^.

**Supplementary Figure 10** provides additional gas exchange parameters, including transpiration rate, stomatal conductance, and leaf temperature. Under red light, the maize plants attained the maximum transpiration rate at around 9100 PAR, about 7.0 mmol m^-^²s^-^¹. Blue light showed a great increase in transpiration up to around 5700 PAR and peaked at approximately 6.4 mmol m^-^²s^-^¹, before it started to decline. Green light was moderately increasing, always below 4.0 mmol m^-^²s^-^¹, while white light had the lowest transpiration rate, peaking at 3.8 mmol m^-^²s^-^¹ at 7100 PAR (**Supplementary Figure 10A**). Under blue light, maize plants had the highest stomatal conductance, reaching a peak of about 200 µmol m^-^²s^-^¹ at 3500 PAR. Under red light, stomatal conductance increased up to 5100 PAR, reaching a maximum of approximately 163 µmol m^-^²s^-^¹, before gradually declining to around 123 µmol m^-^²s^-^¹ at 9100 PAR. Green light showed similar values, stabilizing around 165 µmol m^-^²s^-^¹, while white light consistently demonstrated the lowest stomatal conductance, with a maximum of about 135 µmol m^-^²s^-^¹ (**Supplementary Figure 10B**). Under blue light, leaf temperature increased steeply, reaching a maximum of about 45°C at 7600 PAR. The red and white light showed a similar trend, peaking at around 38°C and 35°C at 9100 and 8250 PAR, respectively. Green light consistently showed the lowest leaf temperature, reaching a maximum of about 27°C at around 4000 PAR (**Supplementary Figure 10C**).

### Boosting maize CO_2_-assimilation by supplementing white light with green light at high irradiance levels

**Figure 4:**
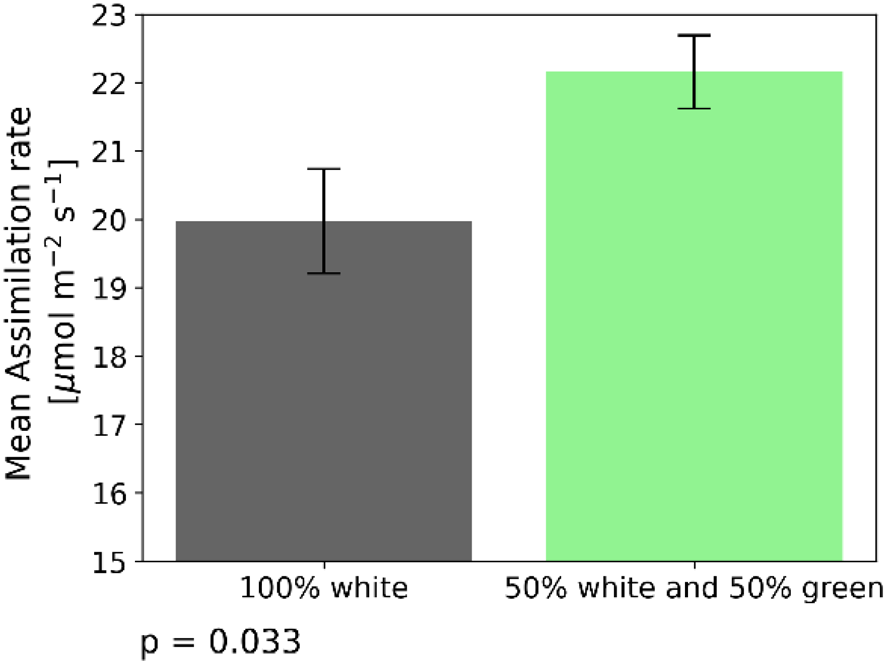
Assimilation rate under 2 different light treatments when recording steady state assimilation rate with the GFS-3000 (Heinz Walz GmbH, Germany) and 20-day-old dark-adapted maize plants (Zea mays saccharata). The duration of the light conditions was set to 60 min. The gray bar indicates assimilation rates under white light (2000 PAR). The green bar depicts assimilation rate under 2000 PAR composed of 50% white and 50% green light. Number of samples: n=10 for each light treatment. t-test p-value = 0.033.

As depicted in **Figure 3B**, exposure to green light at irradiance levels of 2000 PAR and higher, significantly increased assimilation rates. To further study the effects of green light, we compared the assimilation rate under white light (2000 PAR) and a mixture of 50% white light and 50% green light (2000 PAR as well). Assimilation rate for 2000 PAR white light was 19.5 + 1.1 µmol m^–2^s^–1^. Exposed to the light mixture revealed 22.2 + 0.81 µmol m^–2^s^–1^ which is an increase by 14% (**Figure 4**). The difference between these two treatments was verified by a two-sample independent t-test, resulting in a p-value of 0.033.

### Beyond monochromatic light: a DOE-approach

**Figure 5:**
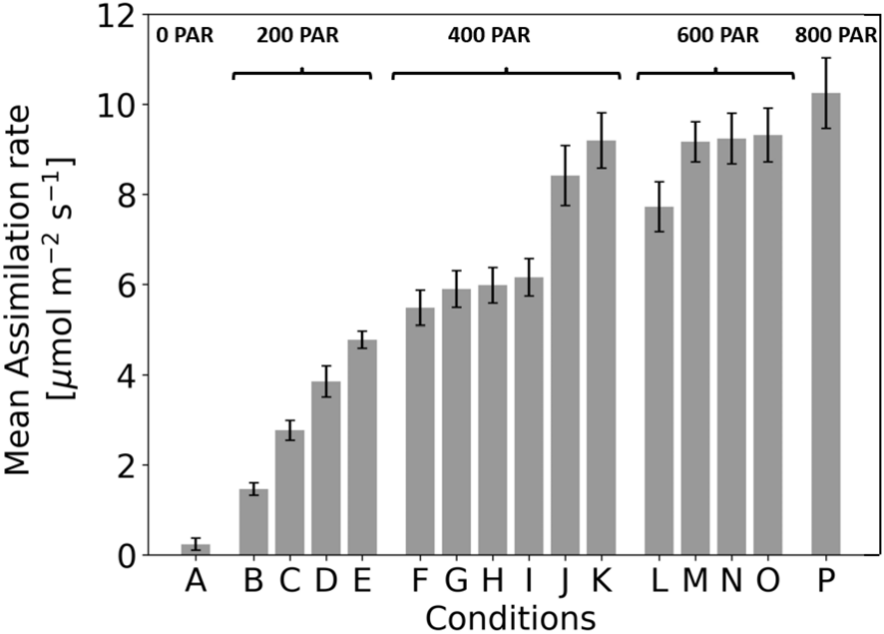
Assimilation rate under 16 different light treatments when recording steady state assimilation rate with the GFS-3000 (Heinz Walz GmbH, Germany) and 20-day-old dark-adapted maize plants (Zea mays saccharata). The duration of the light conditions was set to 30 min. Number of samples: n=20 for each light treatment, every time in a different randomized order. To determine the conditions under which photosynthetic rate varies significantly, ANOVA was performed (for p values, see figure.

In the initial phase of research, the process of photosynthesis was investigated exclusively under conditions of monochromatic light. Subsequently, a comprehensive full factorial Design of Experiments (DOE) methodology was employed to methodically assess the impact of diverse light spectra on the photosynthetic process. An enumeration of the assorted light spectra derived from the DOE is depicted below (**Table 1**). Each lighting condition underwent evaluation through 20 distinct measurements (**Figure 5**), with each iteration featuring a randomized sequence of conditions.

**Table 1:** Detailed factorial design to investigate photosynthesis under various light conditions [A-P].

condition	450nm	465nm	550nm	630nm	PAR
A	–	–	–	–	0
B	–	–	x	–	200
C	x	–	–	–	200
D	–	–	–	x	200
E	–	x	–	–	200
F	x	–	x	–	400
G	–	–	x	x	400
H	x	–	–	x	400
I	–	x	x	–	400
J	x	x	–	–	400
K	–	x	–	x	400
L	x	–	x	x	600
M	x	x	x	–	600
N	–	x	x	x	600
O	x	x	–	x	600
P	x	x	x	x	800

Each letter represents a combination of light wavelengths (450 nm, 465 nm, 550 nm, 630 nm), where ‘x’ stands for wavelengths switched on and ‘-’ for wavelengths switched off. Each active wavelength contributes 200 PAR to the total intensity of photosynthetically active radiation, which is summed up in the last column.

At 200 PAR, the assimilation rates for conditions B, C, D, and E were 1.47 ± 0.14 µmol m^-^²s^-^¹, 2.77 ± 0.22 µmol m^-^²s^-^¹, 3.89 ± 0.34 µmol m^-^²s^-^¹, and 4.78 ± 0.19 µmol m^-^²s^-^¹, respectively. All conditions differed significantly from each other (p<0.001). The increasing assimilation rates (B → C → D → E) reflect a clear dependence on the applied wavelengths. Green light (550 nm) in condition B resulted in the lowest assimilation, while blue light (450 nm) in condition C showed a higher assimilation rate than green. Red light (630 nm) in condition D further increased assimilation compared to 550 nm or 450 nm. The highest assimilation at 200 PAR was observed at 465 nm in condition E.

At 400 PAR, conditions J and K differed significantly from all others (p<0.001). No significant differences were observed between F–G (p=0.121), F–H (p=0.059), G–H (p=0.740), G–I (p=0.356), and H–I (p=0.541). The increasing assimilation rates reflect the additive effect of wavelengths: condition F (450 nm + 550 nm) resulted in moderate assimilation, while G (550 nm + 630 nm) and H (450 nm + 630 nm) showed slightly higher rates. Condition I (465 nm + 550 nm) produced further increases, while the blue combined wavelengths in condition J (450 nm + 465 nm) showed a marked improvement. Condition K (465 nm + 630 nm) yielded the highest assimilation in this PAR range.

At 600 PAR, the assimilation rates for conditions L, M, N, and O were 7.72 ± 0.57 µmol m^-^²s^-^¹, 9.16 ± 0.42 µmol m^-^²s^-^¹, 9.24 ± 0.53 µmol m^-^²s^-^¹, and 9.32 ± 0.62 µmol m^-^²s^-^¹, respectively. Condition L differed significantly from M, N, and O (p<0.001), while no significant differences were observed between M–N (p=0.800), M–O (p=0.643), and N–O (p=0.829). The lower assimilation rate for condition L, which combines 450 nm, 550 nm, and 630 nm, contrasts with the higher rates for M, N, and O. These three conditions include 465 nm, suggesting that this additional blue band enhances assimilation at 600 PAR. The lack of significant differences between M, N, and O indicates that variations in the wavelength combinations at this intensity have minimal impact on assimilation rates.

When comparing 400 PAR to 600 PAR, it is notable that some combinations at 400 PAR (e.g., condition K: 9.19 µmol m^-^²s^-^¹) do not differ significantly from conditions at 600 PAR (e.g., condition M: 9.16 µmol m^-^²s^-^¹). This suggests that the combination of 465 nm and red (condition K) is particularly effective, producing assimilation rates comparable to those at 600 PAR with multiple wavelengths. Adding more wavelengths at 600 PAR (e.g., in condition M) results in only minimal improvements.

When comparing 600 PAR to 800 PAR, conditions M, N, and O (600 PAR) do not differ significantly from condition P (800 PAR, p>0.05). Despite the higher light intensity at 800 PAR, it has no significant effect on assimilation (10.249 ± 0,806 µmol m^-^²s^-^¹), suggesting that assimilation may be saturated and cannot be further increased by additional light intensity. The effect of wavelengths remains consistent, with conditions containing 465 nm achieving the highest assimilation rates.

**Figure 6:**
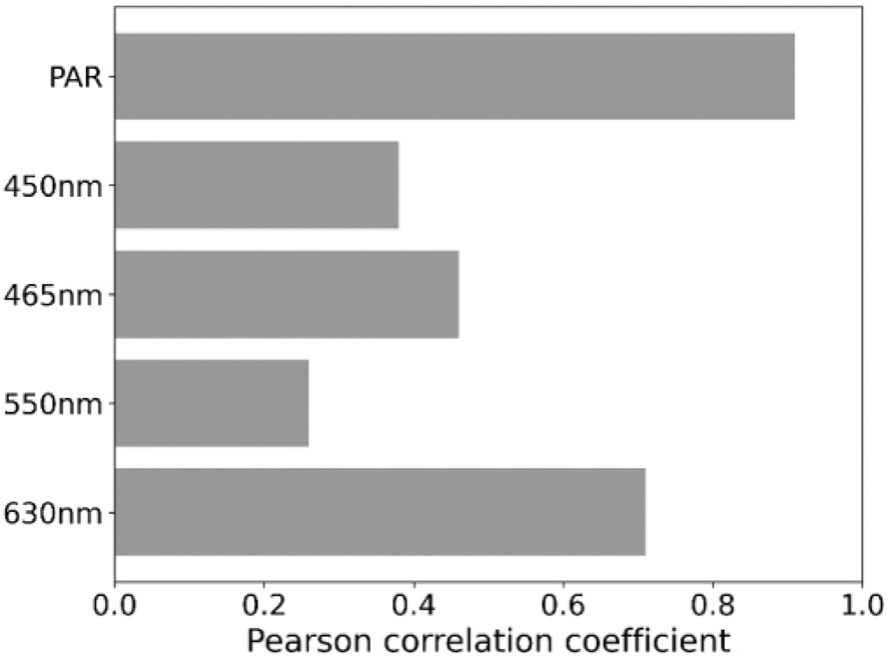
Pearson Correlation Coefficients for Photosynthetic Rate Across Different Wavelengths and PAR Values.

The DOE analysis showed a strong positive link between the PAR value and photosynthesis rate, with a Pearson correlation coefficient of 0.91. Specifically, red light at 630 nm significantly boosts photosynthesis in this PAR range, indicated by a correlation coefficient of 0.71. Blue light has a positive though moderate impact with coefficients of 0.46 for its general impact and 0.38 at 450 nm. Green light shows the least positive correlation with a coefficient of 0.26, suggesting it’s less influential on photosynthesis rates in this range **Figure 6**.

### Chlorophyll fluorescence under high light intensities: exploring maize photosynthesis dynamics

**Figure 7:**
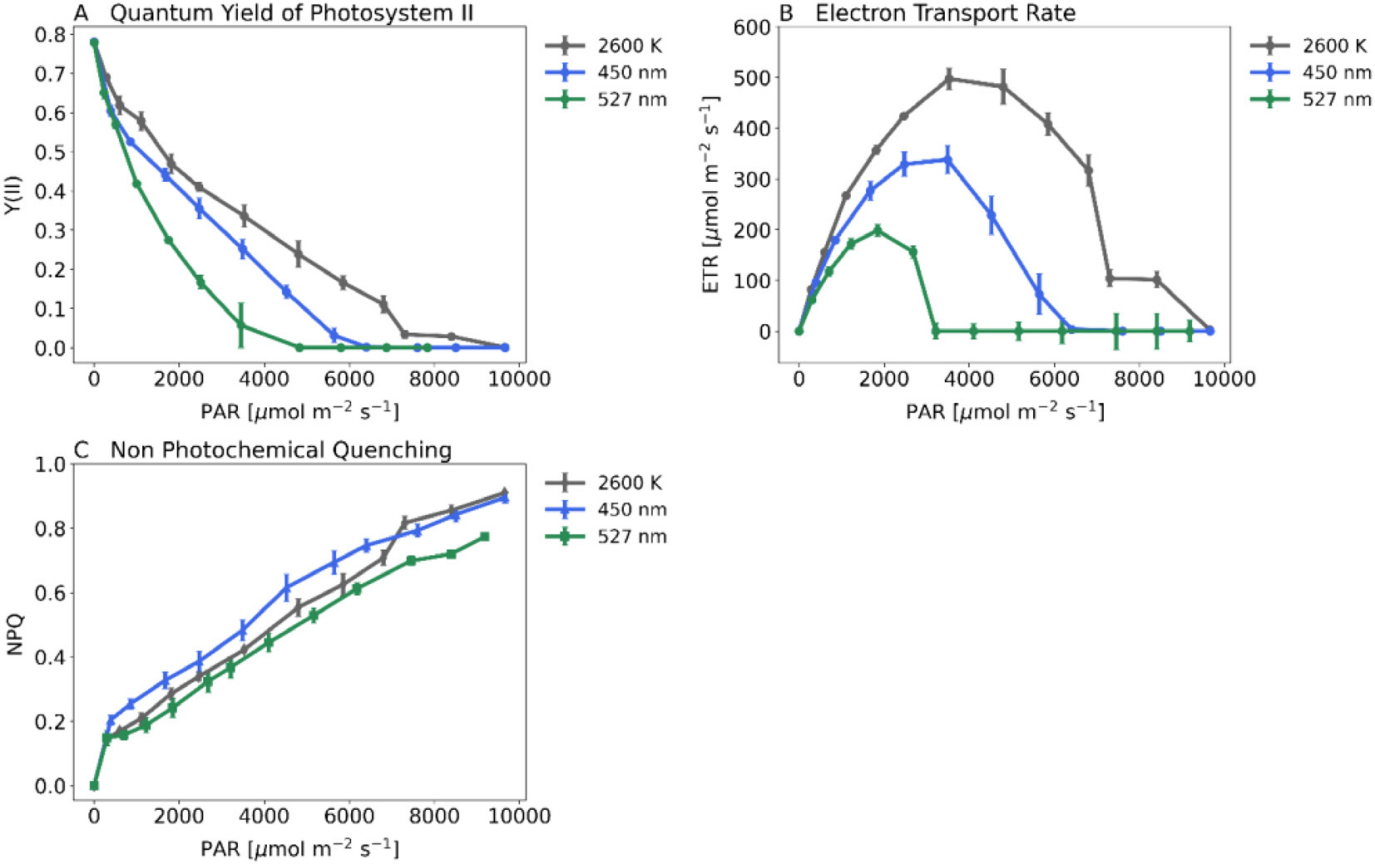
Key PAM-Parameters when recording steady state light response curves with the Imaging-PAM (Heinz Walz GmbH, Germany) and 20-day-old dark-adapted maize plants (Zea mays saccharata). Abscissa: PAR-Level [µmol m^–2^s^–1^]. First stage of the Light Response Curve was set for 60 min. All further stages were each 15 min long. Gray curve: 2600 K, blue curve: 450 nm and green curve: 527 nm. **(A)** Quantum Yield of Photosystem II, **(B)** Electron Transport Rate, **(C)** Non Photochemical Quenching. Number of samples: n=10 for each light treatment.

In addition to gas exchange, we used Imaging-PAM (Heinz Walz GmbH, Germany) to measure Quantum Yield of Photosystem II (Y(II), **Figure 7A**), Electron Transport Rate (ETR, **Figure 7B**) and Non-Photochemical Quenching (NPQ, **Figure 7C**) of maize plants. Quantum yield decreases significantly under green light, being less significant under blue light. Plants under white light show highest ETR reaching 500 µmol m^–2^s^–1^. Plants under blue light reach 350 µmol m^–2^s^–1^ or 200 µmol m^–2^s^–1^ under green light. Non-photochemical quenching did not differ between plants under white or green light below 7000 PAR. However, plants show a significantly higher NPQ under blue light.

### Green light: effects on growth, plant morphology and health

**Figure 8:**
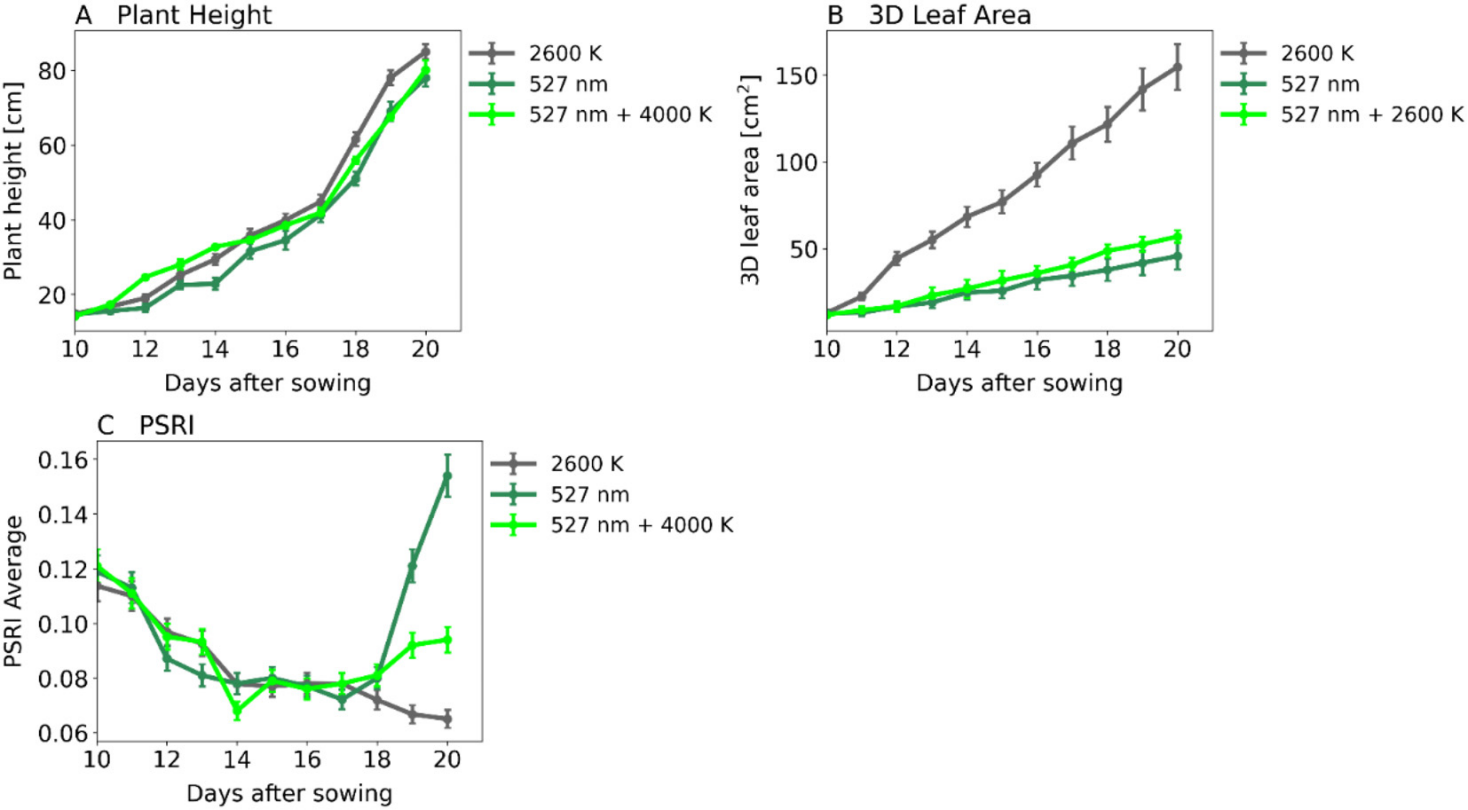
Plant morphology and health. A: Plant Height in cm, B: 3D Leaf area in cm^2^ and C: Plant Senescence Reflectance Index (PSRI). Abscissa: time after sowing in days. 2000 PAR white light (2600 K, gray curve), 2000 PAR 50% white light (2600 K) combined with 50% green light (527 nm, light green curve) or 2000 PAR monochromatic green light (527 nm, dark green curve). **(A)** Plant Height, **(B)** 3D Leaf Area, **(C)** Plant Senescence Reflection Index. Number of samples: n=3 for each light treatment.

**Figure 9:**
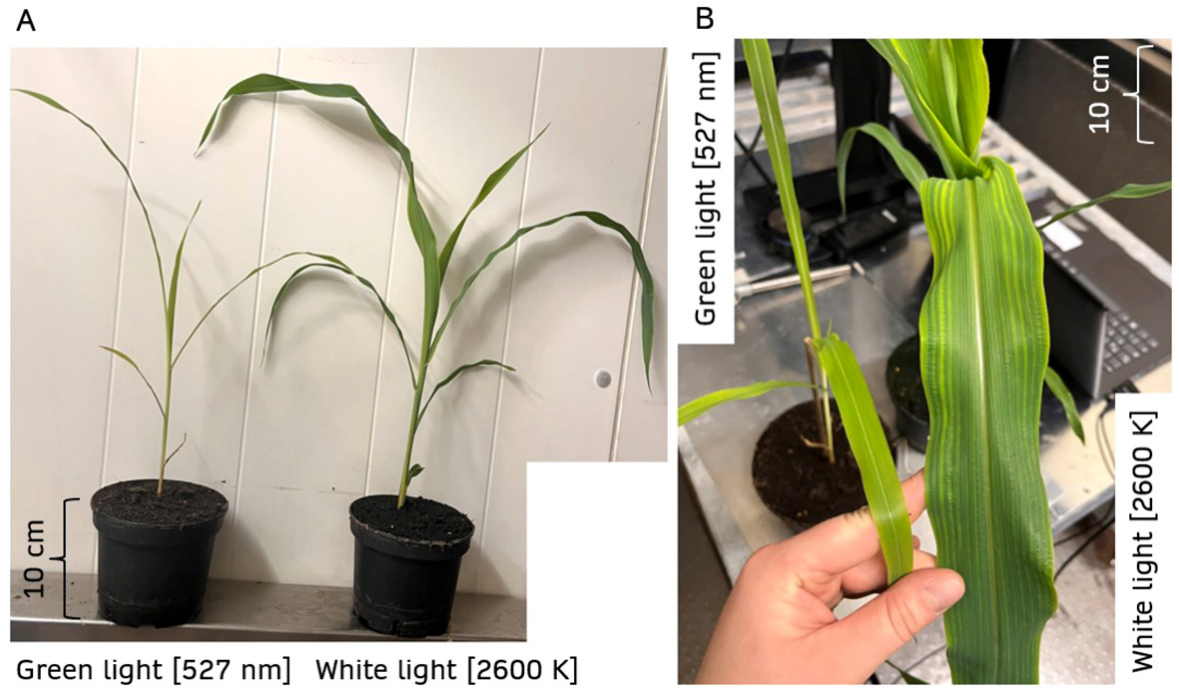
Photographs of 17-day-old maize plants. One of them was grown under monochromatic green light, the other one under white light. The plants show identical plant heights in both treatment groups **(A)**, but the 3D leaf surface area is four times larger under warm white light compared to green light **(B)**. For comparison, scales with a length of 10 cm are provided in the image.

At irradiance levels of 2000 PAR, as shown in **Figures 3**, **4**, the assimilation rate can be increased by supplementing green light. We cultivated plants under 3 light spectra: 2000 PAR white light, 2000 PAR green light and 2000 PAR consisting of 50% white light and 50% green light, respectively. We assessed plant morphology and health using Phenospex PlantEye. Results showed no significant difference in plant height (**Figures 8A**, **9A**) but in 3D leaf area (**Figures 8B**, **9B**). White light-grown plants had 155 cm^2^ 3D leaf areas, while green light-grown plants only reached 45 cm^2^. Plants grown under the white-green spectra reached 56 cm^2^. The Plant Senescence Reflectance Index (PSRI) was used to monitor plant senescence and health status. The PSRI initially rose to around 0.12 and then declined to around 0.08. It continued to decline marginally for plants exposed to white light after 20 days but increased steeply from day 18 after sowing for plants under green light, exceeding the initial threshold of 0.12 (**Figure 8C**).

## Discussion

Using recently developed high intensity LED light sources we were able to observe plant photosynthesis and growth under conditions that correspond to approximately 5 times the intensity of sunlight. Light intensity range was from 5000 PAR for green up to 9500 PAR for red light.

### Light saturation and photoinhibition in maize under varying light conditions

At low light levels, the photosynthetic rate increases with light intensity. As anticipated CO_2_ assimilation attains a saturation point and beyond it, higher light levels do not enhance photosynthetic efficiency (Wang et al., 2012). At high light intensities, Maize reached light saturation points depending on applied light conditions: white light at 4800 PAR, red light at 4600 PAR, blue light at 2300 PAR, and green light at 3700 PAR. According to previous studies, the light saturation point of maize was determined at 1400 to 1600 PAR (Li et al., 2007; Wang et al., 2012; Tt et al., 2020). Multiple reasons might contribute to the observed different light saturation points. Different maize varieties were included in the trials, and distinct genotypes may exhibit divergent responses to elevated light intensities. Zhu et al. used variety *Zea mays* L. Dica 517, Li et al. used two varieties *Zea mays* L. ZD 958 or ZJ 2. Moreover, the light sources were different. Zhu et al. used the LI-6400/XT light source, Wang et al. utilized the LI-6200 light source, while we irradiated the plants with innovative Sevenengine chip modules. Wang et al. measured the gas exchange on the first, fully developed leaf. This is the fourth leaf counted from the top of the plant. Zhu et al. chose three independent, fully developed leaves per plant. We always measured on the uppermost, fully developed leaf. While we allowed the plants to acclimate for more than an hour and then exposed them to each light intensity for 12 minutes, Zhu et al. chose only 15 minutes acclimatization time and 3 minutes per light intensity.

Also, plant cultivation was essentially different. Wang et al. employed a greenhouse, but with distinct temperatures and photoperiods compared to our procedure. Zhu et al. used maize plants grown in the field and thus exposed to much more variable conditions.

At elevated light intensities, photoinhibition may occur, resulting in a reduction of the photosynthesis rate due to excessive light causing damage to the photosynthetic apparatus (Greer and Hardacre, 1989). To our knowledge, light intensities up to 9500 PAR were not investigated so far. Our work reveals that the increasing light intensity raises the rate of CO_2_ assimilation in maize continuously until around 7000 PAR under white light. After that, a decrease in CO_2_ assimilation was observed. This may be related to photoinhibition.

In maize, high light photosynthetic performance is achieved as in other C4 plants by distinctive anatomical and biochemical characteristics. Spatialization of the first step of carbon fixation in mesophyll cells and localization of the Calvin cycle in bundle sheath cells reduces photorespiration and allows maize to retain a high photosynthetic efficiency under conditions where in general C3 plants suffer from carbon loss (Leegood, 2002). Moreover, maize plants have high levels of photosynthetic enzymes, i.e., Rubisco and PEP carboxylase that allow prolonged carbon fixation under high light levels (Wasilewska-Dębowska et al., 2022). The position of chloroplasts in bundle sheath cells is another reason for the high light efficiency in maize (Retta et al., 2023), including the efficient capture of absorbed light and the minimized photochemical loss of energy (i.e., light dissipation). In addition, maize can perform high-level non-photochemical quenching (NPQ), an adaptive protective process which dissipates surplus energy in form of heat, preventing photoinhibition (Ferguson et al., 2023). At ultra-high light irradiances this ability probably postpones the onset of photodamage. Maize and supposedly also other C4 plants can perform photosynthesis and assimilate CO_2_ at these extreme light levels.

### Photosynthetic response to different light spectra

Under blue light, the assimilation rate peaked at about 1800 PAR, then decreased sharply, falling below other light qualities at higher PAR. The results of Le et al. (2021) demonstrated that blue light at 1500 PAR achieved the highest photosynthetic rate. However, this applied to plants that had been cultivated for four weeks under blue light with a wavelength of 450 nm (Le et al., 2021). The plants investigated in our study were all grown under white light.

For red light, the peak was at 4000 PAR, about 40% higher than blue light, and declined slowly, remaining at moderate levels up to 6000 PAR. Green light induced highest assimilation rates, peaking at 3000 PAR and staying steady up to 4000 PAR prior to gradual decline. Our studies confirmed the positive effects of green light on photosynthesis at 2000 PAR using a 50:50 combination of white and green light. Unexpectedly, we observed that maize continued to exhibit photosynthetic activity even at very high intensities up to 8500 PAR, which had not been reported so far for this plant species. Previous studies on the reaction of maize photosynthesis to light intensities just covered the range up to 2000 PAR (Dwyer and Stewart, 1986; Hugh, 2003; Pengelly et al., 2011).

### Impact of green light on photosynthetic efficiency

It has been assumed that green light plays a minor role in photosynthesis, as it is reflected stronger by leaves than red or blue light. However, Gitelson & Merzlyak demonstrated, as early as 1996, that an increase in chlorophyll-a concentration is inversely proportional rather than directly proportional to the reflection of green light (Gitelson and Merzlyak, 1996). Virtanen et al. (2022) confirmed this and showed that leaves with lower chlorophyll concentrations reflected green light significantly than leaves with higher chlorophyll content of the same species. They also concluded that chlorophyll does not reflect light at all. Instead, plant leaves appear green because green light is less efficiently absorbed by chlorophyll a and b compared to red or blue light, thus having a higher likelihood of being diffusely reflected by cell walls (Virtanen et al., 2022). In fact, only 10-50% of green light seems to be reflected by the chloroplasts, while the remaining green light is either absorbed or transmitted to other areas of the plant (Nishio, 2000; Terashima et al., 2009). Chlorophyll a and b maximally absorb in the red and blue range of the light spectrum, but *in vivo* chlorophyll is always associated with other pigments and cellular structures such as flavonoids, anthocyanins, and carotenoids, which modify absorption in the green region of the spectrum (Smith et al., 2017). Carotenoids primarily serve two functions: light harvesting and photoprotection (Gao et al., 2021; Zhang et al., 2022). The ability of green light to penetrate deeper into the leaf tissue has been well-documented (Liu and van Iersel, 2021). The optical density of leaves is primarily determined by tissue morphology and the concentration of photosynthetic pigments (Cutolo et al., 2023). Since chlorophyll a and b absorb mainly in the blue and red range, more green photons can penetrate the mesophyll and be absorbed in deeper leaf layers (Evans and Anderson, 1987; Sun et al., 1998; Brodersen and Vogelmann, 2010). Light scattering in the leaf tissue also creates the so-called ‘detour effect,’ which extends the light path of green light through the leaf profile, giving photons a higher chance of hitting chloroplasts on their way (Vogelmann T, 1989). Arsenault et al. (2020) demonstrated that under high illumination, green light drives photosynthesis more efficiently than red light. Their study, using polarization-dependent two-dimensional electronic-vibrational spectroscopy, revealed that green light contributes to photosynthesis in LHCII, with the mixed vibronic Qy-Qx states playing a crucial role (Arsenault et al., 2020). Gitelson et al. (2021) further suggested that the energy contribution of green light can be comparable to that of red light, and its quantum yield, calculated based on absorbed light, is also comparable (Gitelson et al., 2021). However, the results of Liu and van Iersel showed that at low light intensity, green light had the lowest photosynthetic efficiency, which is attributed to its lower absorption (Terashima et al., 2009). In contrast, under high light intensities, our studies demonstrated that maize attained optimal photosynthetic rates at 4000 PAR under green light, with reduced photodamage compared to blue or red light at equivalent intensities. Another study on *Helianthus annuus* found that supplementing white light with monochromatic green light (λ550 ± 30 nm) increased leaf photosynthesis more efficiently than supplementation with monochromatic red light (λ641 – 690 nm) (Terashima et al., 2009). This aligns with our findings, where a 50:50 application rate of white and green light also enhanced overall photosynthetic rates. Notably, maize exhibited the highest photosynthetic rates under monochromatic green light at intensities between 3000 and 4000 PAR, which exceeded those observed under similar intensities of red or blue light.

### Impact of light intensity on photosynthesis across multiple wavelengths

Since it is well known that monochromatic light conditions do not cover the photosynthetic absorbance spectrum (Li and Liu, 2020), an experimental design was implemented with combinations of four wavelengths of 200, 400, 600 and 800 PAR (O’Carrigan et al., 2014; Yang et al., 2017; Zhang et al., 2019). Pearson correlation coefficients of assimilation rate, PAR level and the effect of wavelengths were examined. The Pearson coefficient quantifies the strength of the linear relationship between two variables (Schober et al., 2018). The results indicated that between 0 and 800 PAR, the assimilation rate correlated most with total light intensity, indicating that the photosynthetic rate increased with light intensity regardless of the wavelength. This strong correlation suggests that light intensity is the key driver of photosynthesis, independent of the specific light quality (Rodriguez-Morrison et al., 2021; Shafiq et al., 2021). This observation is consistent with the findings of Sun et al. (2024), who reported near-linear increases in A within the range of 0 to 300 PAR, with responses varying depending on wavelength (Sun et al., 2025). Similarly, Choi et al. (2021) demonstrated that photosynthesis rates in tomato plants increase under PAR intensities ranging from 200 to 800 μmol m^-^²·s^-^¹, demonstrating a linear trend in the photosynthetic response at lower intensities (Choi et al., 2021). The second most important effect on photosynthesis in this par range was red light.

### Impact on maize growth

We used innovative lighting methods to analyze the effects of light spectra on maize photosynthesis. While green light with intensities over 2000 PAR significantly increased photosynthesis, potentially indicating higher growth rates, it did not correlate with optimal plant growth. For growth studies, three light conditions were applied including 12:12 light/dark cycle and 2000 PAR. White light, monochromatic green light, and a 50:50 mixture of green and white light were used for comparison, as monochromatic light was found unsuitable for maize cultivation (**Supplementary Figure 13** ).

Surprisingly, plant height was similar under all light conditions. However, leaf surface area was only one-fourth under green light compared to white light. Maize plants grown under white light had the best PSRI values, indicating good health. Plants under the green-white combination were healthier than those under monochromatic green light but still less than those under white light. Kaiser et al. (2019) also reported that the combined effects of a light spectrum on plant physiology and growth exceed the sum of the individual wavelengths’ effects on photosynthetic efficiency (Kaiser et al., 2019). These results highlight the need to understand why conditions that significantly increase photosynthesis rates do not promote optimal growth in maize.

Monochromatic green light may cause a mismatch between carbon assimilation and nutrient uptake. It can impair root development, reducing nutrient absorption efficiency. This limits the transport of carbohydrates to the roots, disrupting energy for nutrient absorption (Taiz et al., 2023). As a result, ion channels and transporters for nitrogen, phosphorus, and potassium function less effectively, leading to inadequate nutrient uptake despite enhanced photosynthesis, which hinders biomass conversion and growth.

In monochromatic light, hormone signals necessary for growth may be lacking, affecting development despite high photosynthesis. Cao et al. (2022) demonstrated that monochromatic light influences hormonal signaling pathways, particularly brassinosteroid-mediated developmental processes (Cao et al., 2022). Di et al. (2020) showed that monochromatic light alone is insufficient to support optimal hormonal signaling for plant growth (Di et al., 2021). Auxins, crucial for cell elongation, are regulated by blue light, and phototropins also respond to this spectrum (Darwin, 1880; Keuskamp et al., 2011; Moni et al., 2015). The red-to-blue light ratio also influences cytokinin production (Kara et al., 1997; Pashkovskiy et al., 2021). When exposed to monochromatic green light, these hormonal processes may become disrupted, leading to impaired growth.

Plants have protective mechanisms to dissipate excess light energy. These mechanisms include non-photochemical quenching, the xanthophyll cycle, state transitions and ROS scavenging (Mohr and Schopfer, 1978). However, under monochromatic light, these mechanisms may fail to sufficiently compensate for the accumulation of excess light energy, resulting in cell damage and limited growth. Trojak and Skowron (2021) showed that monochromatic light spectra, such as red and blue light, impair NPQ regulation and antioxidant enzyme activity, leading to excess light energy accumulation and oxidative stress (Trojak and Skowron, 2021).

Our data show that NPQ, triggered by high light, is less efficient under green light. The xanthophyll cycle, primarily activated by blue light (Latowski et al., 2004), may not efficiently convert violaxanthin to zeaxanthin, leading to excess light energy and ROS accumulation.

Additionally, ROS signals under green light may be too weak to trigger sufficient antioxidant enzyme responses, such as superoxide dismutase and catalase.

We are the first to investigate how such high light intensities affect the photosynthetic mechanisms in maize. It was discovered that photosynthesis continued at levels up to 9500 PAR, this had not been examined before. Despite this enhancement in photosynthesis, we did find an obvious discrepancy between high assimilation rates and growth, suggesting that severe irradiance could be compromising nutrient uptake and biomass production.

## Data Availability

The raw data supporting the conclusions of this article will be made available by the authors, without undue reservation.
