# Supplementary material for: Effects of high light intensity and spectral variability on maize photosynthesis and growth
Source: Front Plant Sci. 2025 Mar 26;16:1511768. doi: 10.3389/fpls.2025.1511768 (PMC11978832; doi:10.3389/fpls.2025.1511768)
Supplement: Supplementary file 1 [file DataSheet1.pdf]

Supplemental Material

Maize Gas Exchange Parameter Under Varying Light Conditions

Transpiration rates and stomatal conductivity were enhanced under blue light, reduced under low white light, and similar under green or red light up to 4000 PAR (Figure 10A and Figure 10B). The leaf temperature exhibits a notable variation under blue light (Figure 10C).

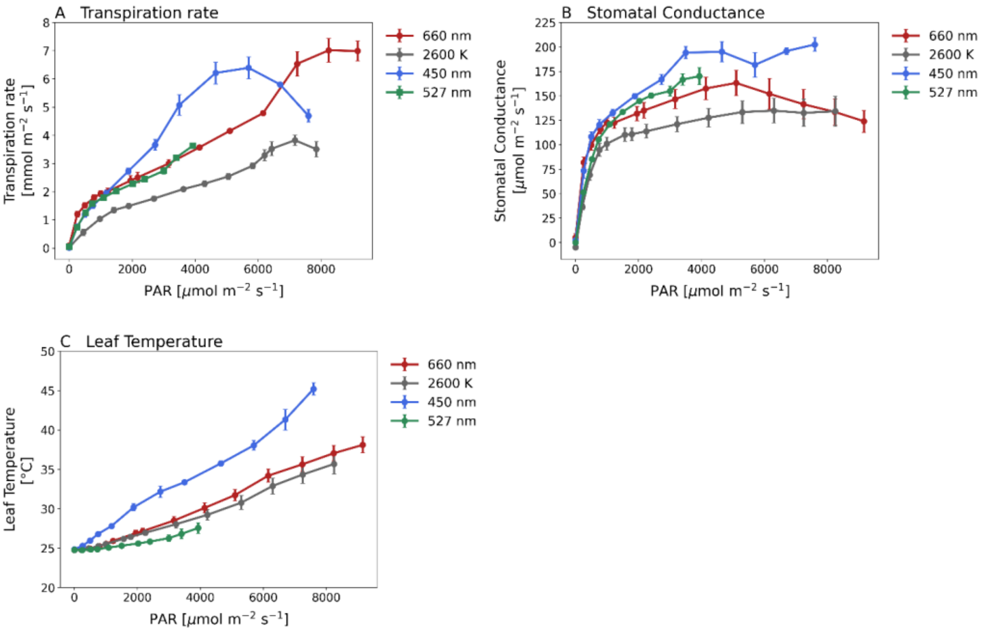

Figure 10 Transpiration rate (A), Stomatal Conductance (B) and Leaf Temperature (C) for four different light treatments at steady state of light response curves in 20-day-old dark-adapted maize plants (Zea mays saccharata). Abscissa: PAR-Level [µmol/m2s]. The first stage of the Light Response Curve was set for 75 min. All further stages were each 12 min long. Red curve: 660 nm, gray curve: 2600 K, blue curve: 450 nm and green curve: 527 nm. Number of samples: n=10 for each light treatment.

Light Responses in Maize: LRC and SPM Methods in PAM Measurements

The PAM measurements in Figure 7 were carried out using light response curves (LRC) under stable conditions. This means that until they reached a stable condition at each stage, the plants were progressively acclimated to varying light levels. In contrast, the single plant measurements (SPM) in Figure 11 show data taken from a single plant without prior adaptation to different light intensities. As there is no stepwise adaptation to the light conditions, the plant may be more stressed at higher intensities, resulting in lower measured values.

When interpreting the results in Figure 7, it should therefore be considered that the LRC method allows more robust measurements by adapting the plants to different light intensities, while the SPM reflects the immediate response to light without an adaptation phase.

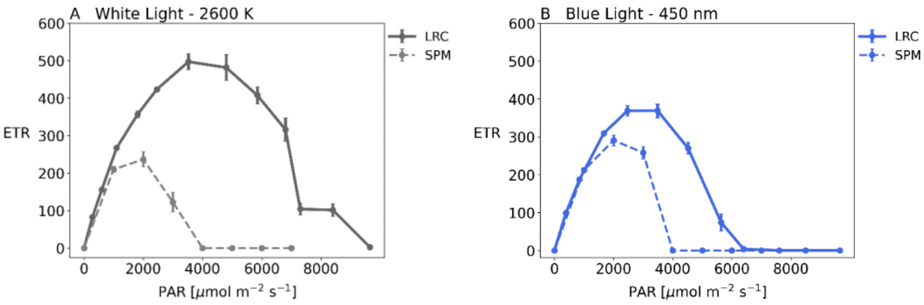

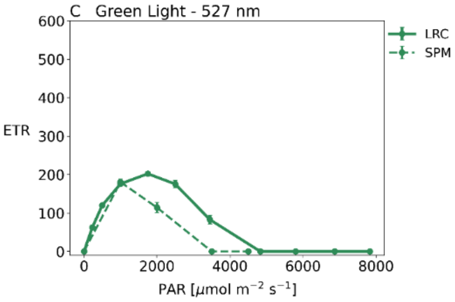

Figure 11 Electron transport rate (ETR) in dark-adapted plants as influenced by the level of PAR (abscissa) under three distinct light treatments: (A) White Light (2600 K), (B) Blue Light (450 nm), and (C) Green Light (527 nm). 'LRC' denotes 'Steady State-Light Response Curve,' representing the response where a dark-adapted plant was acclimated to successive light intensities incrementally. In contrast, 'SPM' stands for 'Single Plant Measurement,' depicting the response of a new, dark-adapted plant exposed for 60 min to each specific light intensity for PAM measurements. For a detailed explanation of these methodologies, refer to the 'Methods' section.

Validating Steady State in Maize: 60-Minute Y(II) Response to Different Light Conditions

The purpose of selecting a time duration of 60 minutes for data collection in Figure 3 and 7 to ensure the attainment of steady state conditions. Figure 12 shows the temporal variation of photosystem II quantum yield (Y(II) after 60 minutes irradiation. The plants were exposed to three irradiation conditions: white light (2600 K), blue light (450 nm), or green light (527 nm). They underwent a 60-minute period of dark adaptation before exposure to light with the specified wavelength and intensity. Under 1000 PAR, maize plants exhibit greater values when exposed to green light or blue light compared to white light. Under blue and green light with 3000 PAR, plants do not achieve a Y(II) above 0. However, with white light, plants are still able to reach a Y(II) above 0, even at 3000 PAR.

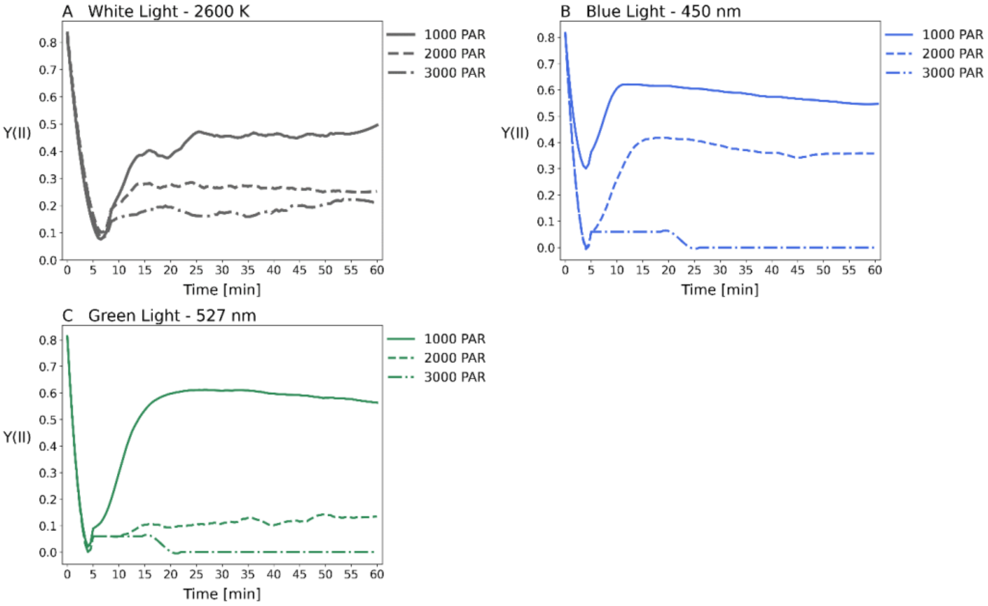

Figure 12 Quantum yield of photosystem II of dark-adapted, 20-day-old maize plants (Zea mays saccharata) as a function of time (abscissa) for 1000 PAR (-), 2000 PAR (- -) and 3000 PAR (-.-). (A) White light (2600 K), (B) Blue light (450 nm), (C) Green light (527 nm). Plants were exposed to the respective light intensity for 60 min.

ANOVA Results for Figure 5

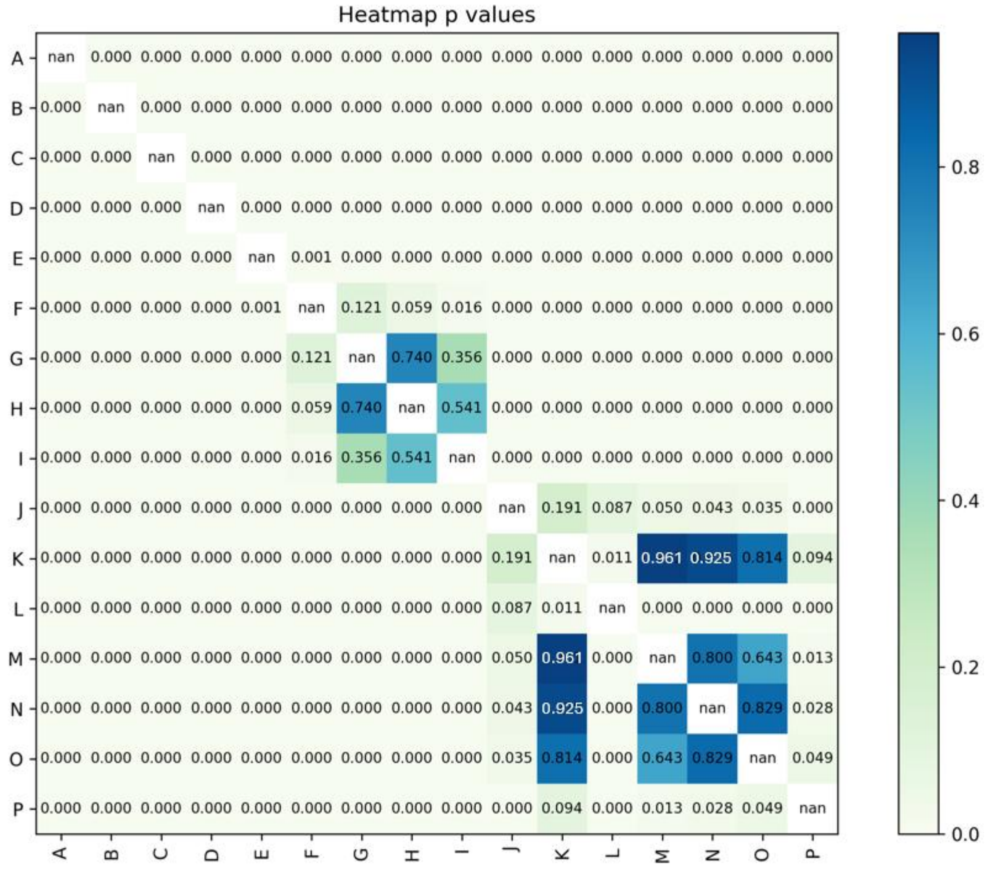

Figure 13 Heatmap of p values from the ANOVA to evaluate the significance of the assimilation rate across the 16 conditions presented in Figure 5.

Growth under monochromatic green light

Initially, we examined the growth of maize plants under white light and compared it to growth under monochromatic green light. The plants were positioned under the designated light conditions immediately after seeding and cultivated for a duration of 10 days. Exposed to white light, the plants exhibited typical, robust growth (Figure 14A), whereas those cultivated under monochromatic green light were stunted (Figure 14B).

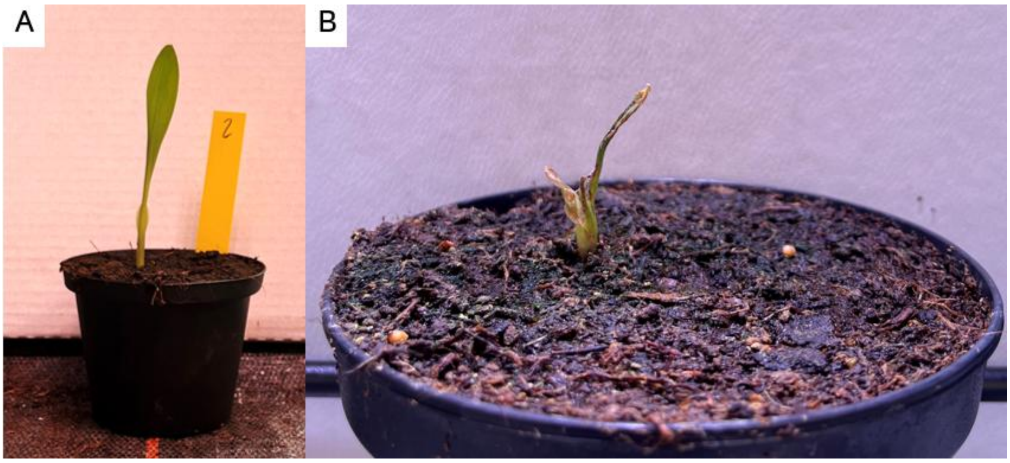

Figure 14 10-day-old maize plants cultivated under white light (A, 2600 K) or monochromatic green light (B, 527 nm) since sowing. Under both conditions, the plants were lighted in a 12h:12h rhythm with a light intensity of 2000 PAR.
